# Enhanced Micromixing Using Surface Acoustic Wave Devices: Fundamentals, Designs, and Applications

**DOI:** 10.3390/mi16060619

**Published:** 2025-05-25

**Authors:** Jin-Chen Hsu

**Affiliations:** Department of Mechanical Engineering, National Yunlin University of Science and Technology, Douliou 64002, Yunlin, Taiwan; hsujc@yuntech.edu.tw

**Keywords:** surface acoustic wave, interdigital transducer, acoustic mixing, microfluidics, micromixer

## Abstract

Microfluidics-based mixing methods have attracted increasing attention due to their great potential in bio-related and material science fields. The combination of acoustics and microfluidics, called acoustofluidics, has been shown to be a promising tool for precise manipulation of microfluids and micro-objects. In general, achieving robust mixing performance in an efficient and simple manner is crucial for microfluidics-based on-chip devices. When surface acoustic waves (SAWs) are introduced into microfluidic devices, the acoustic field can drive highly controllable acoustic streaming flows through acoustofluidic interactions with micro-solid structures, which have the advantages of label-free operation, flexible control, contactless force, fast-response kinetics, and good biocompatibility. Therefore, the design and application of various SAW micromixers have been demonstrated. Herein, we present a comprehensive overview of the latest research and development of SAW-based micromixers. Specifically, we discuss the design principles and underlying physics of SAW-based acoustic micromixing, summarize the distinct types of existing SAW micromixers, and highlight established applications of SAW micromixing technology in chemical synthesis, nanoparticle fabrication, cell culture, biochemical analysis, and cell lysis. Finally, we present current challenges and some perspectives to motivate further research in this area. The purpose of this work is to provide an in-depth understanding of SAW micromixers and inspire readers who are interested in making some innovations in this research field.

## 1. Introduction

Microfluidics involves the technology of precisely manipulating fluids at a microscale and has attracted widespread attention for decades [1,2]. Microfluidic technology has a wide range of applications in the fields of chemical and biological engineering, including biochemical analysis [3,4], DNA sequencing [5], sample preparation and analysis [6,7], cell separation and detection [8,9,10], environmental monitoring [11], etc. Compared with macroreaction systems, microfluidic devices in these applications exhibit advantages of low sample and reagent consumption, rapid response time, cost-effective operation, and high throughput [12,13]. In microfluidic systems, micromixing is one of the most common operations for various microanalysis applications [14,15,16,17].

Micromixing specifically refers to uniformly mixing two or more types of fluids together in a microscale microfluidic chip. When applying microfluidics in chemical and biomedical sciences, efficient mixing is essential, which requires robust micromixers. An efficient micromixer can achieve rapid and uniform mixing between samples and provide intimate contact between the reagent molecules, thereby enabling dramatically improved interactions in chemical and biological reactions [18]. Since the characteristic length of the flow channels (typically on the order of 100 μm) in micromixers is shortened to the microscale, the ratio of surface area to volume is greatly increased. This enables great enhancement of heat and mass transfer between fluids and their reactions [19,20]. However, the regime of the fluid flows is laminar in nature with a very small Reynolds number (Re) because the flow rates of the fluids in such microchannels are generally very low [21,22]. For example, in a water-based microfluidic system (a fluid density of 997 kg/m^3^ and a dynamic viscosity of 0.89 mPa s at 25 °C) with a fluid flow rate of 1.0 mm/s, the Reynolds number is of the order of 0.1. Therefore, turbulent mixing does not readily occur, and the mixing of the species is primarily dominated by molecular diffusion, which is an inherently slow process and requires a long microchannel to achieve sufficient mixing [23].

To realize thorough species mixing within short microchannels for microfluidic applications, exploring the use of various energy sources and physical transduction mechanisms to achieve effective and efficient micromixing has been an active research topic [24,25,26]. Depending on the mixing principle employed, micromixers can be categorized as passive micromixers and active micromixers [27,28]. Passive micromixers can restructure (e.g., deflect, fold, and split) the fluid flow to boost molecular diffusion and/or chaotic advection by altering the configurations of microchannels or constructing heterogeneous microstructures in the channels [29,30,31,32]. These actions disrupt the movement between the fluids and can increase the contact area at the fluid interface, thereby improving the mixing efficiency. Passive micromixers have the advantages of requiring no external excitation/source and being easy to integrate with other microfluidic devices. However, passive micromixers still have a few inherent constraints that limit their practicality, including heavy dependence on fluid properties and flow rates, relatively long mixing times required, large footprints, and low flow controllability [33,34]. In particular, for high-viscosity fluids (such as sputum and blood) that are common in bio-related applications, the diffusion mixing time could be significantly prolonged [35,36]. On the other hand, active micromixers use the disturbance produced by an external field, such as pressure, thermal, magnetic, electric, and acoustic-based approaches, to improve the mixing efficiency [37,38,39,40,41,42]. A schematic comparison of passive and active micromixers over typical ranges of mixing efficiency, mixing time, and mixing length, along with potential pros and cons, is shown in Table 1.

Generally, active micromixers have higher mixing efficiency than passive ones. Nevertheless, their integration with microfluidic systems could be more complicated, and the operation of active micromixers requires an external power source. Among these, the electric and magnetic field-assisted approaches usually require labeled fluids, such as electrical polarity or magnetic properties. Therefore, these types of active micromixers could accompany the inevitable restraints of limited applicability and mixing efficiency that heavily depend on the fluid properties. On the other hand, acoustics-integrated microfluidic (i.e., acoustofluidic) mixers have attracted considerable attention due to their unique advantages, such as precise and label-free manipulation, tunable speed, large and contactless force, and good biocompatibility. These attractive features endow acoustofluidics with great potential for addressing many challenges in chemical and biomedical engineering. Table 2 presents a concise comparison that briefly contrasts acoustic micromixers with other types of active micromixers.

**Figure 1:**
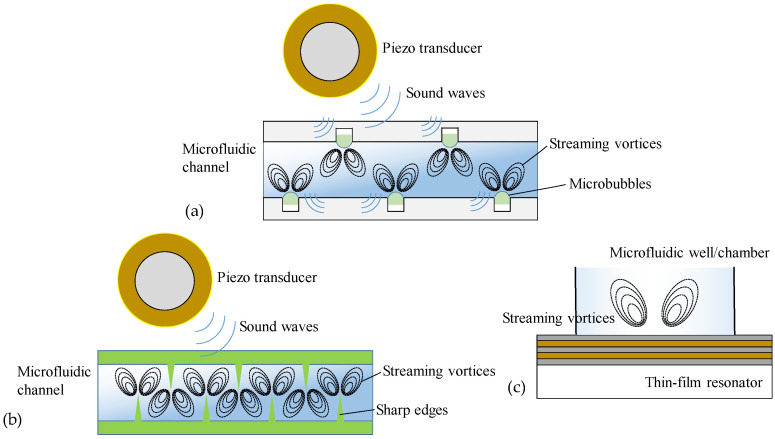
Schematic representation of acoustofluidic micromixers. (**a**) Bubble micromixer. (**b**) Sharp-edge micromixer. (**c**) Resonant acoustic micromixer.

In acoustofluidics-based micromixers, the sound waves (usually at a high frequency) are launched to propagate into fluid media and induce pressure fluctuations to generate disturbance for facilitating mass transport of fluids. So far, there are several distinctive classes of acoustic micromixers: bubble, sharp-edge, surface-acoustic-wave (SAW), and resonant acoustic micromixers (see schematics in Figure 1) [45,46,47,48,49,50]. Although each type of active micromixer has its own set of benefits, they also have drawbacks that restrict their practical use. The bubble micromixer uses sound waves to drive the surface of deliberately created microbubbles inside the channel to enhance mixing. Although effective, bubble micromixers are usually limited by the inconvenient bubble-trapping process and unstable bubble structure during operation. Sharp-edge micromixers employ the tip oscillations of sharp-edge structures inside the microfluidic channel (including sidewall wedges, sidewall rods, off-sidewall sharp edges, and inlet conjunction needle). Upon electrically actuating an external acoustic transducer (commonly operating at several kHz), the sharp edges can be acoustically oscillated to generate a pair of counter-rotating vortices (i.e., acoustic streaming) that break the interface of laminar flows around their tips to dramatically facilitate mass transport and intensive mixing of fluids. However, sharp-edge micromixers usually require a higher voltage threshold to activate sufficient tip oscillation, a lower flow rate to maintain stronger acoustic streaming, and an appropriate applied frequency to meet the specific oscillation frequency of the sharp edges. Otherwise, the mixing efficiency will not be sufficiently enhanced. Therefore, careful consideration should be given to the rational design of sharp-edge microchannels and optimization of operating parameters to obtain efficient sharp-edge micromixers. Resonant acoustic micromixers use a piezoelectric layer or membrane underneath a microchannel to launch upward propagating bulk acoustic waves (BAWs) into the fluids, where BAWs are generated through the longitudinal or flexural resonances of the piezoelectric layer/membrane (multilayered or suspended). Such BAW devices work as resonators and can exhibit a high-quality factor at the resonant frequency, resulting in a larger initial acoustic amplitude. Due to viscous dissipation, part of the energy of the BAWs in the fluids is converted into kinetic energy, thus generating acoustic streaming and facilitating micromixing. Generally, this type of acoustic micromixer operates at very high frequencies (ranging from hundreds of MHz to several GHz), so the fluids can be exposed to the acoustic field for many oscillating cycles in a short period of time and mixed ultrafast. Due to the large attenuation of ultrahigh-frequency acoustic waves, their working distance in fluids is usually short, making them suitable for mixing at the length scale below 100 μm. The main disadvantage of resonant acoustic micromixers is that BAW resonators require complex and elaborate design and fabrication procedures. SAW micromixers utilize the distinctive acoustic modes that travel along the surface of an elastic medium, which, when applied to microfluidics, have typical wavelengths in the range of 10–100 μm and amplitudes on the order of nanometers [51]. The most common type of SAWs is known as the Rayleigh wave, which comprises both longitudinal and transverse vibrations, oscillating in an elliptical trajectory. However, there are many other types of SAWs, such as shear-horizontal (SH) SAWs, Scholte waves, Love waves, Sezawa waves, pseudo-SAWs, and leaky SAWs, which may be used in microfluidic applications in lieu of the Rayleigh waves [52,53,54]. SAW micromixers generally consist of one or more interdigital transducers (IDTs) patterned on a piezoelectric substrate. When an alternating voltage is applied to the IDTs at the resonant frequency, SAW is generated, and its energy can be radiated into the fluids placed in the wave-propagation path. This radiation arises from the mismatch of sound velocities between substrate and fluids, and the leakage of the radiation in the fluids can generate a pressure wave to drive acoustic streaming [55]. Compared with other acoustic micromixers, SAW micromixers have the advantages of simple structure, high precision, diverse modalities, and a wide available frequency range (from tens of MHz to several GHz). By introducing additional innovative design considerations, extensive studies have shown that SAW micromixing technology can easily achieve high mixing efficiency, high controllability, and high tuning flexibility, making it dominant in acoustic micromixers. However, the thermal impact of SAW on the fluids also produces a heating effect and temperature rise, which is very crucial for microfluidic applications [56]. Keeping in view the importance of these facets, many research investigations have been conducted in the past decade to advance the development of SAW micromixers and deepen the understanding of their working mechanisms. Therefore, it makes it imperative to review the previous literature related to SAW micromixing technology.

This review article aims to provide a systematic overview of recent advances in SAW-based acoustofluidic mixer systems for microfluidics applications. Specifically, it is organized as follows. Firstly, we start by discussing the fundamental principles necessary for the consideration and design of SAW micromixers. Then, we summarize the established SAW micromixer models that classify different SAW field excitations with different IDT configurations (including single straight IDT, single non-straight IDT, and multiple IDTs), where we highlight the challenges of SAW-based mixing approaches in microfluidic systems and provide insights to guide future studies in this area. Finally, we illustrate state-of-the-art micromixing applications using the SAW platform and highlight the unprecedented opportunities offered by SAW micromixers and SAW-related acoustofluidics in a wide range of chemical and biomedical engineering applications, including chemical synthesis, nanoparticle fabrication, cell culture, biochemical analysis, and cell lysis.

## 2. Fundamentals

### 2.1. Mixing Behavior in Microfluidic Environments

Although fluid flow in both macroscale and microscale environments is governed by the same physical laws, microfluidic devices are not simply miniature versions of their macroscale counterparts. Fluid dynamics generally divide fluid flows into two flow regimes: laminar flow and turbulent flow [57]. At the macroscale level, mixing is conventionally achieved by a turbulent flow, which allows the segregation of the fluid in small domains, resulting in increased contact surface area and shortened mixing paths. However, the fluid flow regime in microchannels is typically laminar due to the small dimensions of channels and low fluid flow velocity, where the fluid streams flow parallel to each other and the velocity at any location within the fluid stream is invariant with time. This implies that advection occurs only in the direction of the fluid flow. Consequently, mixing of fluids in microchannels relies primarily on slow molecular diffusion rather than on the rapid convective mass transfer processes that dominate in turbulent systems.

In microfluidics, two dimensionless numbers are commonly used to quantitatively characterize the flow mixing: Reynolds number (Re) and Péclet number (Pe) [58,59]. The Reynolds number, which represents the ratio of inertial forces to viscous forces within a fluid, is defined as:

(1) Re=ρvDhμ=vDhν,

where *ρ* is the fluid density, *μ* is the dynamic viscosity, *ν* is the kinematic viscosity, *v* is the average flow velocity, and *D_h_* is the hydraulic diameter of the channel. In channel flow, the onset of turbulence can be predicted by the Reynolds number. The transition Reynolds number between the well-defined regimes of laminar and turbulent flow is usually expected to be in the range of 1500 to 2300. For most microfluidic systems, Reynolds numbers well below the transition value are ubiquitous, where viscous effects dominate inertial effects so that hydrodynamic instabilities do not emerge. The Péclet number represents the ratio of the rate of advection to the rate of diffusion and is defined as:

(2) $$\mathrm{Pe}=\frac{vL}{D},$$

where *L* is the characteristic length, and *D* is the mass diffusion coefficient. A high Péclet number means the diffusion rate is much slower than the advection rate, so the latter of the two phenomena predominates the mass transport. However, introducing a high advection rate into the microfluidic system is insufficient to enhance mixing. As long as the Reynolds number remains low, such that turbulence is absent, any further increases in advective transport simply serve to increase the mixing length only. The key to promoting effective mixing is to instead introduce chaotic advection to disrupt the laminarity of the flow [60]. Due to the low Reynolds numbers at the microfluidic scales, the generation of turbulence to affect mixing is equally difficult. Relying on diffusion for passive mixing usually leads to long mixing times when working on the microscale. On the other hand, the same fluid recirculation generated using electrohydrodynamics and SAWs can be used to drive chaotic advective transport to achieve effective micromixing.

### 2.2. SAW Devices

**Figure 2:**
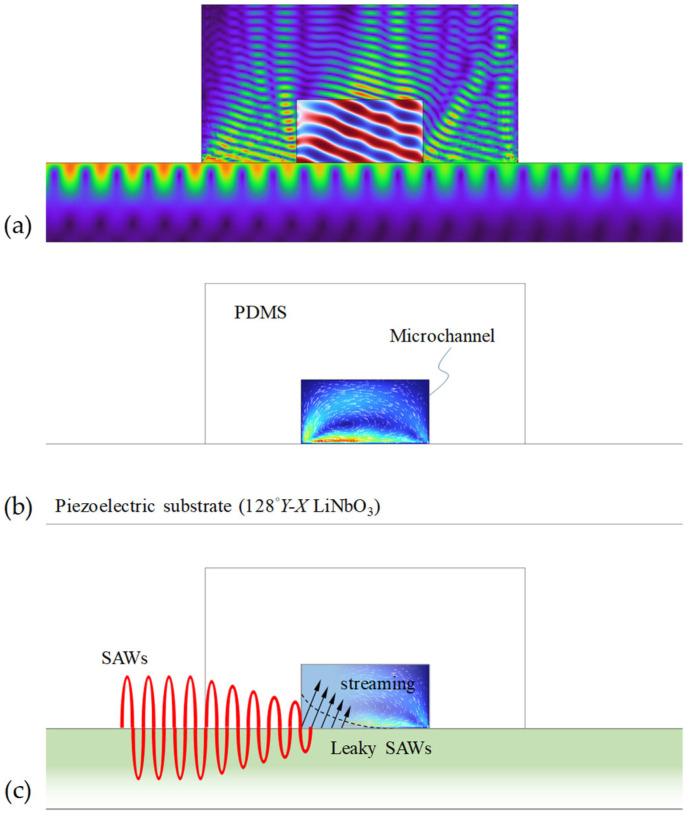
Simulated results illustrating the acoustic process of SAW-based micromixer. (**a**) The SAWs propagate along the substrate surface and transform into leaky SAWs at the solid–fluid interface, radiating the acoustic pressure into the microfluidic channel at the Rayleigh angle. (**b**) Leaky SAWs drive bulk recirculation (acoustic streaming flow) in the microchannel. (**c**) Schematic illustration showing the energy of the SAWs radiating into the microfluidic channel.

SAW technology involves a piezoelectric substrate with IDTs deposited on its surface. Figure 2 shows the schematics of the mixing mechanism in a typical SAW-based micromixing device. When an alternating current (AC) or radio-frequency (RF) signal is applied to the electrodes of the IDTs, it generates an oscillating electric field. This electric field causes the piezoelectric substrate to deform due to the piezoelectric effect, generating elastic waves that propagate on its surface. Since most of the elastic energy is confined adjacent to the substrate surface (within a few wavelengths), SAWs are attractive for providing very efficient fluid–structural coupling and, thus, are an efficient mechanism for driving microfluidic processes. Furthermore, the elastic energy present at high frequencies due to the SAW is responsible for extreme accelerations [61]. For example, the acceleration induced by SAW with a frequency of 10 MHz and an amplitude of 1 nm can reach 10^6^ m/s^2^. For this reason, the SAW method has been widely adopted to address fundamental constraints in microfluidics.

The material chosen for the substrate of a SAW device should possess high electromechanical coupling (*K*^2^), allowing it to efficiently convert electrical energy into mechanical energy (elastic waves) and vice versa. Crystals like quartz (SiO_2_), lithium-niobate (LiNbO_3_), and lithium-tantalate (LiTaO_3_) are common piezoelectric materials used in SAW devices [62]. However, in microfluidic applications, LiNbO_3_ with the specific orientation of 128° *Y*-rotation, *X*-propagation cut is suitable and most commonly adopted to generate Rayleigh SAW due to its high electromechanical coupling (*K*^2^~5.6%), transparency, biocompatibility, and chemical stability.

In a piezoelectric material, the elastic field and the electromagnetic field are coupled. Under a quasi-static assumption, the constitutive equations governing the piezoelectric motion, written in terms of the tensor components, can then be written as [63,64]

(3) $$\begin{array}{l} T_{ij}=c_{ijkl}S_{kl}-e_{kij}E_{k}\\ D_{i}=e_{ikl}S_{kl}+\varepsilon_{ik}E_{k} \end{array},$$

where *T_ij_* and *D_i_* are the components of the Cauchy stress tensor (second rank tensor) and electric displacement vector, respectively, *S_kl_* and *E_k_* are the components of the elastic strain tensor (second rank tensor) and electric field vector, respectively, *e_ikl_* is the component of the piezoelectric stress tensor (third rank tensor), *ε_ik_* is the dielectric constant (second-rank tensor), and *c_ijkl_* is the elastic stiffness constant (fourth-rank tensor). An orthogonal Cartesian coordinate system is used where $i,j,k,l\in \left\{1,2,3\right\}$ and the Einstein summation convention over repeated subscripts is assumed. The coordinate directions such that $\left(x,y,z\right)\equiv \left(1,2,3\right)$ are also assumed. The quasi-static approximation allows the electric field to be determined by $E_{k}=-\partial_{k}\varphi$, where *φ* is the electric potential field, and $\partial_{k}$ denotes a partial derivative operation with respect to the coordinate direction *k*.

The relationship between stress and elastic displacement for acoustic wave propagation in a piezoelectric solid is governed by Cauchy’s equation of motion as follows [65]:

(4) $$\partial_{i}T_{ij}=-\rho_{s}\omega^{2}u_{j},$$

where *ρ_s_* is the mass density of the piezoelectric solid, *u_j_* is the elastic displacement, and *ω* is the angular frequency. Simultaneously, the gradient of the electric displacement obeys Gauss’s law. In the case of the piezoelectric solid with no free-electrical charge, Gauss’s law can be expressed as [65]:

(5) $$\partial_{i}D_{i}=0.$$

The relationship between the elastic strain and displacement caused by the small acoustic disturbance can be linearized as

(6) $$S_{kl}=\frac{1}{2}\left(\partial_{k}u_{l}+\partial_{l}u_{k}\right).$$

Equations (3)–(6) form a complete set for the analysis of acoustic wave propagation in a piezoelectric solid. The solution for a SAW mode can be determined by adequately satisfying elastic and electric boundary conditions on the surface. The equations combined with the boundary conditions can be solved using the finite-difference time-domain (FDTD) method, finite-element analysis (FEA), direct closed-form analysis, and hybrid methods combining closed-form and numerical techniques [66,67,68]. Assuming time harmonic excitations, the displacement and electric potential fields of the obtained SAW mode can be generally characterized by the following form

(7) $$\begin{array}{l} u_{j}=A_{j}e^{-i\left(kx-\omega t\right)}e^{-\alpha kz}\\ \varphi =A_{4}e^{-i\left(kx-\omega t\right)}e^{-\alpha kz} \end{array},$$

where *A_j_* and *A*_4_ are the amplitudes of the displacement vector and electric potential, respectively, *k* is the wavenumber, *α* is the attenuation coefficient of the SAW amplitude along the *z*-direction (the depth direction of the piezoelectric substrate), and *t* denotes time. The propagation velocity and wavelength of SAW can then be determined by $c_{\mathrm{SAW}}=\omega /k$ and λ=2π/k, respectively.

There are two important electrical boundary conditions that define two distinctive SAW velocities: open-circuit and short-circuit conditions, corresponding to the surface covered by a hypothetical medium with zero permittivity and covered by a very thin metallic film, respectively. These two electrical boundary conditions produce electrically open and electrically short SAW velocities, denoted as *c*_0_ and *c*_∞_, respectively. By using the Ingebrigtsen approximation, the electromechanical coupling coefficient *K*^2^ can then be calculated by [69]

(8) $$K^{2}=2\frac{c_{0}-c_{\infty }}{c_{0}}.$$

Equation (8) indicates that the greater the difference between the two distinctive SAW velocities, the higher the electromechanical energy conversion rate. In SAW devices, electric potentials are applied to the electrodes of the IDTs to generate the SAWs. The electric potentials on the charged and grounded electrodes are typically provided as [70]

(9) $$\varphi_{c}=V_{0}e^{i\omega t},\;\varphi_{g}=0.$$

where *V*_0_ is the voltage amplitude of the applied electric potential.

### 2.3. Configurations and Materials of IDTs

For SAW micromixers, the methods of generating various liquid streaming through the SAW devices are crucial. This can be effectively achieved using IDTs in the SAW devices, which are introduced and discussed in this subsection. When designing a SAW device, its intended function and application must be considered. The choice of IDT material and configuration has a considerable impact on the excitation of the SAW field and the ultimate performance of the device. The other key design parameters for the IDTs of a SAW device include resonant frequency (or called center frequency), SAW velocity, *K*^2^ coefficient, substrate material (including crystal cut orientation and propagation direction), finger width and finger spacing, number of finger pairs, and acoustic aperture (the overlapping length of finger electrodes). These key parameters are fundamental and may determine the primary response of the excited SAWs. More advanced considerations involve frequency spectrum, bandwidth, power-output density, dispersion, anisotropic effects of the piezoelectric substrate, various propagation losses, and impedance matching. A well-designed IDT can significantly improve SAW generation efficiency, reduce insertion loss, suppress noise signals, and reduce signal distortion.

**Figure 3:**
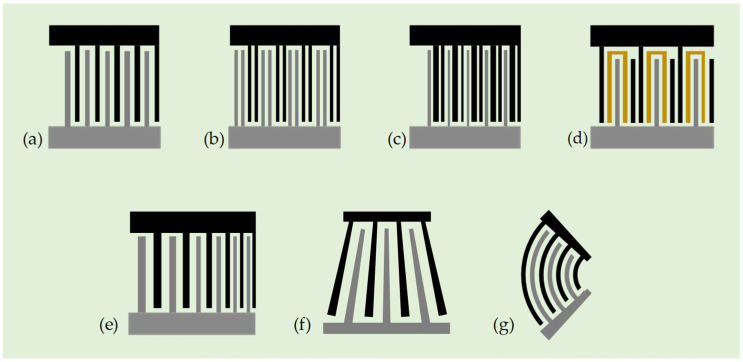
Various IDT designs: (**a**) standard bidirectional straight IDTs, (**b**) split IDTs, (**c**) single-phase unidirectional transducer (SPUDT), (**d**) floating electrode unidirectional transducer (FEUDT), (**e**) chirped IDT, (**f**) slanted-finger IDT (SFIT), and (**g**) focused IDT.

To better understand the IDT features, Figure 3a shows a schematic diagram of a standard uniform IDT consisting of two electrode fingers, bus bars, and an electrode pad. It is characterized by equal width and spacing of the fingers, where the width and spacing of the fingers determine the spatial period of the wave and, thus, the wavelength of the SAW. Since the SAW wavelength is equal to the electrode period of this IDT type, the inter-electrode interaction generates in phase-scattered waves, which results in a stronger internal reflection and spurious modes. This problem can be addressed using split electrode IDTs, as shown in Figure 3b. A more critical drawback is known as the bidirectional effect, where this type of IDT generates two SAW beams propagating in opposite directions. Therefore, half of the SAW energy may be wasted in one direction, resulting in a lower power efficiency for acoustic streaming applications. Despite these unfavorable issues, standard uniform IDT is still widely adopted in acoustofluidic systems due to its simplicity. On the other hand, various different types of IDTs, as shown in Figure 3c–g, have been further explored to pursue enhanced and/or novel acoustofluidic functionalities in different contexts and targets, including unidirectional IDTs, chirped IDTs, slanted finger IDTs (SFITs), and focused IDTs [71,72].

A. Unidirectional IDTs: Unidirectional IDTs are designed to address the bidirectional effect and transmit SAW energy primarily in one direction. Several different IDT configurations can be used to achieve a unidirectional function. The most commonly used are the single-phase unidirectional transducers (SPUDTs). It can reduce bidirectional loss and minimize triple transmission echo in SAW devices. Enhancing the unidirectionality is also found to be an effective way to suppress insertion loss. There are several SPUDT cell designs, in which the electrode-width-controlled (EWC) cell is the best known. A traditional EWC-SPUDT cell consists of three finger electrodes (as shown in Figure 3c) [73], two adjacent narrow electrodes, and one wide electrode, whose widths are *λ*/8 and *λ*/4, respectively. The gap distance between the two adjacent narrow fingers is *λ*/8, and that between the narrow finger and the wide finger is 3*λ*/16. However, there are other design versions of the electrode width and gap distances for an EWC-type SPUDT. Another type of unidirectional IDT employs floating electrodes, as shown in Figure 3d, which is called floating electrode unidirectional transducers (FEUDTs) [74]. The floating electrodes are not connected to any electric potential and do not contribute to the generation of the wave. Instead, they are used to minimize the insertion loss by lowering electrode resistance, reducing parasitic capacitance, improving wave propagation, and providing better impedance matching.
B. Chirped IDTs: This type of IDT can generate broadband response by linearly or nonlinearly modulating the width and spacing of the interdigital fingers, as shown in Figure 3e [75]. This geometric modulation yields a gradient change in the finger pitch, which can generate SAWs with different resonant frequencies and considerably enlarge the bandwidth. The broadband characteristics of the chirped IDTs enable precise control of the SAW propagation properties, such as the wavelength of the excited SAWs. Typically, chirped IDTs require sophisticated design and fabrication techniques. However, they have the advantage of being able to customize the spectrum profile or compensate for dispersion effects caused by substrate materials or device geometry. Chirped IDTs are particularly well suited for manipulating droplets and particles in stationary fluids with an additional degree of freedom by continuously varying or tuning the operating frequency. The frequency tunability of chirped IDTs may also be suitable for adapting wavelength-sensitive fluid manipulation in microstructures.
C. SFITs: They can also be used to produce a broadband response similar to that of a chirped IDT, allowing the SAW to be tuned over a range of frequencies. However, the difference is that the period of the electrodes is changed by tilting their arrangement, rather than modulating the width and spacing of the fingers along the transverse direction [76]. Therefore, the IDT has a fan-shaped or tapered configuration, as shown in Figure 3f. The advantage of this configuration is that the position of the excited SAW beam can be controlled depending on the frequency. As with the chirped IDTs, this design may also require complex optimization and fabrication processes. By tailoring the angle of the IDT fingers and optimizing the slant geometry, the desired bandwidth and transduction characteristics of SAWs can be achieved.
D. Focused IDTs: Unlike previous IDT designs, focused IDTs (as shown in Figure 3g) utilize curved electrode fingers to concentrate acoustic energy into a narrow beam, pinpointing a small focal point [77]. It is often used in acoustofluidic micromixers to improve mixing efficiency by utilizing its higher acoustic power intensity. The curved electrode fingers may be in the shape of a simple circular arc. However, since many crystal cuts of piezoelectric materials are strongly anisotropic, some of the literature suggests designing the curved shape of focused IDTs based on the concentric wave surface (slowness surface) so that the angle-dependent SAW energy velocity is precisely directed to the focal point, and the resulting curve may differ from an exact circular arc shape [78,79].

Apart from the piezoelectric material of the substrate, the electrode materials of IDTs also influence the performance and electromechanical coupling coefficient of SAW devices [52,80]. The electrode materials should have high conductivity in order to minimize the series resistance in the transmission of the excitation signal. Furthermore, it is also important to consider the acoustic impedance (*Z = ρv*, in which *ρ* and *v* are the density of the material and velocity of the waves) of the electrode materials, along with their chemical/thermal stabilities, stress, and adhesion to the substrate. Electrodes with a large acoustic impedance help confine the acoustic energy within the piezoelectric substrate. However, a thick electrode may cause a mass-loading effect. For SAW devices in microfluidic applications, aluminum, gold, and copper (Al, Au, and Cu) are the commonly used electrode materials. At frequencies below GHz, Al electrodes have the advantages of low resistivity and low acoustic impedance. However, it has the problems of low mechanical strength, low melting point, and poor electrical corrosion resistance. Therefore, the lifetime of Al electrodes is usually a concern. Au electrodes offer advantages in high power, liquid, or corrosive environments, in addition to characteristics of low resistivity. Au electrodes also have superior anti-oxidation properties at elevated temperatures. However, at higher frequencies, Au electrodes exhibit large mechanical losses, relatively large mass loading, and reflections. Like the Au electrodes, Cu electrodes have a higher reflectivity due to their large mass density. However, the resulting SAW velocity variation with Cu electrodes is quite moderate compared with Au electrodes. In unidirectional transducers, a large and widely varying reflectivity is required through relatively thin electrodes, which makes Cu a suitable electrode material for SPUDTs, in addition to other types of IDTs [80]. Efficient adhesion of Au and Cu electrodes on the piezoelectric substrates is essential to use them in SAW devices. Chromium and titanium (Cr and Ti) are most suitable for use as the adhesion layers. Therefore, in SAW devices, Au/Ti, Au/Cr, Cu/Ti, and Cu/Cr composite electrodes are much more common in practical applications than simple Au and Cu electrodes.

### 2.4. Acoustofluidics and Acoustic Streaming

SAW-based micromixers rely on acoustic streaming driven by SAWs to enhance advective mixing. Acoustic streaming is known as an inherent steady fluid flow driven by the absorption of intense acoustic oscillations in the fluid medium. This phenomenon was first explained by Lord Rayleigh in 1884 [81] and subsequently studied by Nyborg and Westervelt [82,83]. They demonstrated that the streaming flow due to sound-wave propagation can be obtained via the momentum equation of a steady incompressible laminar flow driven by an external body force *F_j_* (also called the acoustic streaming force). Earlier studies have introduced the Reynolds stress obtained from the averaging operation over the Navier–Stokes equations to account for acoustic fluctuations in fluid momentum [84,85]. Accordingly, the time-averaged incompressible viscous momentum equation governing the fluctuating fluid can then be written as

(10) $$\rho {\dot{v}}_{i}+\rho v_{j}\partial_{j}v_{i}=F_{j}-\partial_{i}p+\mu \partial_{i}\partial_{i}v_{j},$$

where *ρ* is the fluid density, *v_j_* is the fluid velocity, and *p* is the fluid pressure. The dot at the top of the variable represents the derivative with respect to time *t*. Nyborg derived the form of the acoustic streaming force *F_j_* as follows:

(11) $$F_{j}=-\rho \left({v^{\prime }}_{k}\partial_{k}{v^{\prime }}_{j}+{v^{\prime }}_{j}\partial_{k}{v^{\prime }}_{k}\right),$$

where *v*′*_j_* represents the time-averaged velocity caused by the acoustic wave displacement. Therefore, the acoustic streaming force can be calculated once the acoustic wave field is known. Physically, when an SAW interacts with a fluid, the SAW with a displacement field shown as in Equation (7) turns into a leaky SAW and radiates longitudinal waves into the fluid at the Rayleigh angle *θ_R_*. The Rayleigh angle referred to is determined by:

(12) $$\theta_{R}=\sin^{-1}\left(\frac{c_{0}}{c_{\mathrm{SAW}}}\right).$$

The analytical form of the leaky SAW displacement field can be expressed as

(13) $$\begin{array}{l} u_{x}=Ae^{-i\left(k_{L}x-\omega t\right)}e^{-\alpha_{L}k_{L}z},\\ u_{z}=-i\alpha_{L}e^{-i\left(k_{L}x-\omega t\right)}e^{-\alpha_{L}k_{L}z}, \end{array}$$

where *A* and *k_L_* are the initial displacement amplitude and complex wavenumber of the leaky SAW, respectively, and *α_L_* is the attenuation coefficient. Equation (13) describes that the leaky SAW decays exponentially with distance from the source due to the attenuation by viscosity and radiation along its transmission through the medium. The attenuation coefficient *α_L_* is determined by

(14) $$\alpha_{L}^{2}=1-\left(\frac{c_{L}}{c_{0}}\right),$$

where *c_L_* and *c*_0_ are the leaky SAW velocity and the sound velocity in the fluid, respectively. Using the relationship ${v^{\prime }}_{j}={\dot{u}}_{j}$, the components of the acoustic streaming force *F_j_* can be rewritten as [84]

(15) $$\begin{array}{l} F_{x}=-\left(1-\alpha_{L}^{2}\right)A^{2}\omega^{2}k_{i}e^{2\left(k_{i}x-i\alpha_{L}k_{i}z\right)},\\ F_{z}=-i\alpha_{L}\left(1-\alpha_{L}^{2}\right)A^{2}\omega^{2}k_{i}e^{2\left(k_{i}x-i\alpha_{L}k_{i}z\right)}, \end{array}$$

where *k_i_* is the imaginary part of the complex wavenumber *k_L_*. The acoustic streaming force *F_j_* acts in the main fluid volume as a body force in Equation (10) and determines acoustic streaming patterns induced by SAWs in the fluid.

It has been reported that two main types of acoustic streaming flows (ASFs) may be responsible for the enhancement of SAW-driven micromixing: Rayleigh–Schlichting streaming and Eckart streaming [86,87]. These two types of ASFs are generated through distinct and complex physical processes, respectively. The Rayleigh–Schlichting streaming is formed by dissipating acoustic energy into the boundary layer along nonslip solid boundaries (e.g., the channel walls or substrate), thereby generating periodic microvortices driven by a viscous boundary layer. These boundary-driven microvortices are confined to short distances from the boundaries (typically less than the acoustic wavelength) and consume a significant amount of power. In contrast, Eckart streaming is formed by dissipating acoustic energy into the bulk of fluid (bulk-driven streaming), creating a spatially dispersed fluid jet that is propelled by an acoustic beam in the propagation direction of acoustic waves. Therefore, Eckart streaming can be generated for a longer acoustic attenuation length.

To address the complexity of ASFs driven by SAWs in microfluidics, Nyborg’s perturbation theory is one of the most commonly used approaches. The theory reduces the Navier–Stokes equations to a set of nonlinear equations coupling the acoustic and flow velocity fields, and allows the physical problems involving acoustic streaming to be solved correctly under relatively simple mathematical formulations [64,88,89,90,91,92]. For a linear viscous compressible fluid, the governing equations include the continuity and momentum equations

(16) $$\begin{array}{l} \dot{\rho }+\partial_{j}\left(\rho v_{j}\right)=0,\\ \rho {\dot{v}}_{j}+\rho v_{l}\partial_{l}v_{j}=-\partial_{i}p\delta_{ij}+\eta \partial_{k}\partial_{k}v_{j}+\left(\mu_{B}+\frac{1}{3}\mu \right)\partial_{j}\partial_{l}v_{l}, \end{array}$$

where *μ_B_* is the bulk viscosity. The constitutive relation of fluid between the pressure and density is provided by *ρc*_0_^2^ *= γp*, where *γ* is the specific heat capacity ratio in the fluid. Using the perturbation method, the fluid pressure, velocity, and density are expanded in the asymptotic series form as

(17) $$\begin{array}{l} p=p_{0}+p_{1}+p_{2}+\cdots ,\\ v_{j}=v_{0j}+v_{1j}+v_{2j}+\cdots ,\\ \rho =\rho_{0}+\rho_{1}+\rho_{2}+\cdots , \end{array}$$

Here, the zeroth-order field represents the equilibrium state, while the first-order and second-order fields represent the fluctuations caused due to acoustic excitation. Substituting Equation (17) into Equation (16), the first-order equations that represent the time-harmonic component of the fluidic response to the acoustic actuation are provided as

(18) $$\begin{array}{l} {\dot{\rho }}_{1}+\rho_{0}\partial_{j}v_{1j}=0,\\ \rho_{0}{\dot{v}}_{1j}=-\partial_{i}p_{1}\delta_{ij}+\eta \partial_{k}\partial_{k}v_{1j}+\left(\mu_{B}+\frac{1}{3}\mu \right)\partial_{j}\partial_{l}v_{1l}. \end{array}$$

Similarly, the time-averaged second-order equations are provided by

(19) $$\begin{array}{l} \langle {\dot{\rho }}_{2}\rangle +\langle \rho_{0}\partial_{j}v_{2j}\rangle =-\partial_{j}\langle \rho_{1}v_{1j}\rangle ,\\ \rho_{0}\langle {\dot{v}}_{2j}\rangle +\langle \rho_{1}{\dot{v}}_{1j}\rangle +\rho_{0}\langle v_{1k}\partial_{k}v_{1j}\rangle =-\partial_{i}\langle p_{2}\delta_{ij}\rangle +\eta \partial_{k}\partial_{k}\langle v_{2j}\rangle +\left(\mu_{B}+\frac{1}{3}\mu \right)\partial_{j}\partial_{l}\langle v_{2l}\rangle , \end{array}$$

where the angle brackets denote the time-average operation over a full oscillation time, and the products of first-order fields act as the source terms in the equations. The formulation demonstrates that the response of a fluid to a harmonic force can be divided into two components with two distinct time scales–the acoustic-driven steady oscillation on the fast time scale described by the first-order equations and the time-averaged motion of the fluid on the slow time scale described by the second-order equations (referred to as the acoustic streaming). For mixing phenomena, the concentration field *C* in the fluid can be described by the classical convective-diffusion equation as follows [93]:

(20) $$\dot{C}+v_{j}\partial_{j}C=D\partial_{k}\partial_{k}C.$$

The typical diffusion time scale is several orders of magnitude slower than the characteristic time of the ASF.

In practice, micromixing efficiency can be evaluated using flow visualization techniques. The mixing index can serve to quantify the efficiency of a micromixer by calculating the ratio between the concentration of species (the grayscale value of pixel intensity between the inlet and outlet) over the cross-section of the channel and the mean value [43,44]. The collected data are converted into a mixing index (*σ*) after calculation by a set of formulas. The details are shown as follows:

(21) $$\sigma =\sqrt{\frac{1}{n}\sum_{i=1}^{n}\left(I_{i}-I_{m}\right)},$$

where *n* is the number of pixels in the cross section, *I_i_* is the grayscale value of pixel intensity, *I_m_* is the mean of the grayscale values of pixel intensity of *n* pixels, and mixing index *σ* is the standard deviation of the intensity distribution across the width of the outlet cross section.

(22) $$\eta =\left(1-\frac{\sigma }{\sigma_{0}}\right)\times 100\%,$$

where *η* is the mixing degree ranging from 0 to 100%, and *σ*_0_ is the standard deviation at the inlet cross section. The mixing degree is directly correlated with the mixing efficiency, i.e., a higher mixing efficiency means better mixing results.

## 3. SAW-Based Microfluidic Mixers

### 3.1. SAWs Excited by a Straight IDT

**Figure 4:**
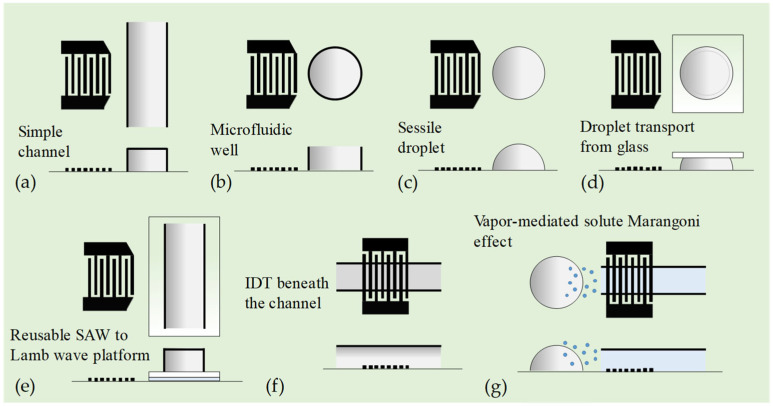
Microfluidic mixing via SAWs generated by single straight IDT. (**a**) A straight IDT generates SAW to drive ASF within a continuous-flow straight microchannel. (**b**) SAWs on a LiNbO_3_ chip to generate fast mixing flows in a microfluidic well [95]. (**c**) Mixing inside a nanoliter sessile droplet using an intense ASF induced by SAWs [96]. (**d**) Mixing nanoliter droplets between a glass and a piezoelectric substrate using SAWs [97]. (**e**) A reusable micromixer platform using SAW device. The anti-symmetric higher-order lamb waves are generated on the thin glass plate to radiate compressional waves into the microchannel to induce fluid motion [98]. (**f**) Acoustofluidic mixing technique inside a single-layered microfluidic channel. High-frequency SAWs generated from the IDT placed right beneath the channel mixed the fluid flow under the influence of strong ASFs [99]. (**g**) SAW-induced heating platform to enhance the vapor-mediated solute Marangoni effect for rapid mixing of sessile droplets [100].

Figure 4 shows different layouts of SAW micromixers using a straight IDT. The straight IDT can generate plane TSAWs covered by the IDT aperture. Figure 4a shows a conventional SAW micromixer, where a straight IDT generates SAW to drive acoustic streaming within a continuous-flow straight microchannel. Hsu and Chang [94] demonstrated that a straight IDT can also launch acoustic plate modes that couple into a SAW device, thereby improving the mixing efficiency. SAW-driven chaotic mixing was quantitatively explained by Shilton et al. [95], who report the use of 20 MHz SAWs on a LiNbO_3_ chip to generate fast mixing flows in a microfluidic well (see Figure 4b). The microfluidic well consisted of a ring with an inner diameter of 2 mm to accommodate 2.5 μL of fluid within the well. They showed that SAWs can drive chaotic flows in the microfluidic well. In general, the larger the acoustic excitation energy, the larger the fluid inertia in the system, which drives chaotic advection because the increase in inertia in the system amplifies the perturbations in the system, promoting random stretching and folding of fluid elements over length scales, leading to the crossing of streamlines. However, the increase in viscosity inherently suppresses chaotic advection and, thus, the mixing effect. They attributed this to nonlinear effects caused by convective acceleration that cannot be ignored in fast-streaming flows induced at high MHz frequencies associated with SAWs.

Shilton et al. [96] also demonstrated mixing inside nanoliter sessile droplets (diameter ≳ 100 μm) using an intense ASF (see Figure 4c). Due to the extremely small droplet size, they employed ultrahigh-frequency (up to 1.1 GHz) TSAWs excited by a straight IDT on a LiNbO_3_ substrate, resulting in acoustic attenuation lengths of approximately 50 μm in the fluid and 20 μm on the substrate, respectively. The diameter of a droplet with a volume of 1.2 nL on the substrate surface is about 100 μm, which is larger than the acoustic attenuation length scale and much larger than the TSAW wavelength of 3.2 μm (1.1 GHz). Under these conditions, compartmentalized three-dimensional vortices can be produced separately in the front and rear parts of the droplet to achieve rapid mixing.

Zhang et al. [97] demonstrated transporting and mixing 2 μL droplets between a glass and a piezoelectric substrate using SAWs with a frequency of 27.5 MHz and an RF power of 28.5 dBm (see Figure 4d). An IDT and reflectors were fabricated on a LiNbO_3_ substrate, and its working surface between the IDT and a reflector was modified to be hydrophobic. Droplets to be transported were first pipetted onto a glass substrate. The glass substrate was adjusted so that the droplets could contact the working surface of the piezoelectric substrate, and then it was moved down. These droplets could be successfully transported from the glass surface to the piezoelectric substrate because of their “adhesion work” difference.

Kishor et al. [98] demonstrated a reusable micromixer platform using an SAW device (see Figure 4e). A Y-shaped microchannel was made from PDMS and bonded to a thin glass plate. It was then coupled to the SAW generated on the LiNbO_3_ substrate using water as an acoustic coupling layer. The anti-symmetric higher-order Lamb waves are generated on the thin glass plate to radiate compressional waves into the microchannel to induce fluid motion. Using an SAW frequency of 50 MHz and at a flow rate of 1 μL/min, a mixing efficiency of 80% was obtained within 1.5 s for an input signal of 50 V.

Ahmed et al. [99] proposed a simple and efficient acoustofluidic mixing technique inside a single-layered polydimethylsiloxane (PDMS) microfluidic channel (see Figure 4f). Their SAW device is composed of a straight IDT, located beneath the PDMS microchannel. The sample and sheath fluids were injected through two inlets of the PDMS microchannel for mixing. High-frequency (140 MHz) SAWs, generated from the IDT placed right beneath the channel, mixed the two fluids under the influence of strong ASFs. The acoustofluidic mixing device realizes complete mixing at a total flow rate of 50 μL/min using an input voltage of 12 V. Later, by numerical and experimental flow visualizations, they observed that the vertically induced acoustic momentum flux was observed to derive a pair of asymmetric micro-vortices that served as an acousto-hydrodynamic barrier for flow control and mixing. This approach was then used to achieve rapid, controlled flow mixing at low power (<6.0 V) and high throughput (~0.2 mL/min) with viscous fluids (~10 mPa⋅s).

Cha et al. [100] proposed an SAW-induced heating platform to enhance the vapor-mediated solute Marangoni effect for rapid mixing of sessile droplets (see Figure 4g). The heater consists of a straight IDT of resonant frequency at 29 MHz deposited on a LiNbO_3_ substrate and a silver nanowire–PDMS composite as an ultrasound absorbing layer. When the IDT is electrically actuated, SAWs are produced and immediately absorbed in the composite layer by viscoelastic wave attenuation. The conversion from acoustic to thermal energy occurs, leading to rapid heating. The heating-mediated enhanced vaporization of a volatile liquid accelerates the solutal Marangoni flows and, thus, enables mixing high-viscosity droplets.

### 3.2. SAWs Excited by a Non-Straight IDT

**Figure 5:**
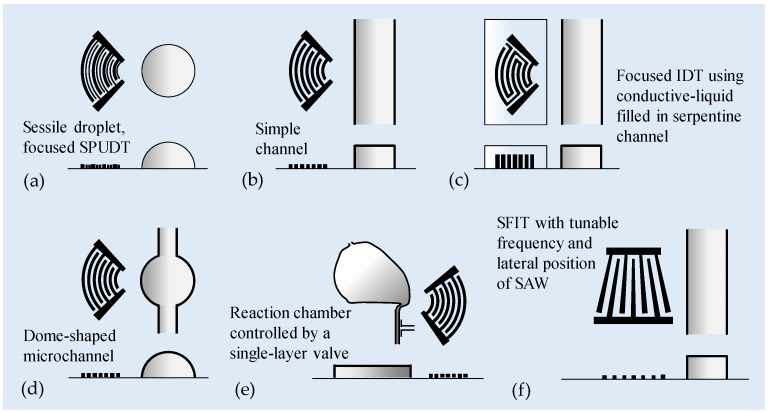
Microfluidic mixing via SAWs generated by single non-straight IDT. (**a**) Using focused SPUDT to generate focused SAWs for intense micromixing in a microliter droplet [101]. (**b**) Focused SAWs launched perpendicular to the flow to drive channel flow mixing [102,103,104]. (**c**) A conductive liquid-based focused SAW (CL-FSAW) device for mixing. The PDMS channel contains electrode channels as focused IDTs for focused SAW generation and a main fluidic channel [105]. (**d**) A dome-shaped chamber-based SAW (DC-SAW) device for mixing [106]. (**e**) A flexible platform for performing various protocols on the chip. The mixing process was enhanced by focused SAWs, and the customized delivery of fluid into the mixing chamber was controlled by a single-layer valve [107]. (**f**) SAWs generated by an SFIT. The lateral position of the excitation SAWs can be fine-tuned by adjusting the frequency, allowing for more precise control of the mixing region [108,109].

Compared to the straight IDTs, focused IDTs can generate focused SAWs with concentrated acoustic energy and induce a larger amplitude and a stronger disturbance. Therefore, the focused IDTs have the capacity to concentrate acoustic radiation at a certain region and efficiently induce internal stirring in a small volume of liquid. Figure 5 shows different layouts of SAW micromixers using focused IDT. Using focused SPUDT to focus the SAWs at a frequency 30 MHz, Shilton et al. [101] demonstrated increased efficiency and speed in generating intense micromixing in microliter droplets (a 0.5 μL dyed water drop pipetted onto a 2 μL drop of transparent glycerin), within which acoustic streaming is induced due to the focused SAW beneath the droplet (see Figure 5a). The used focused SPUDT design combines the focused IDT and SPUDT concept that can generate unidirectional focused SAWs by the superposition of transmitted and reflected SAWs between the IDTs, which can minimize the energy loss. Their results showed that the focused SPUDT generated the higher micromixing intensity due to the more narrowly focused SAW radiation that substantially enhances acoustic streaming in the droplet, increasing the effective diffusivity well beyond the results of the straight SPUDT.

Luong et al. [102] employed SAWs with a frequency of 13 MHz, launched perpendicular to the flow, to demonstrate channel flow mixing. The wave was generated by two designs of IDTs on LiNbO_3_ substrate: straight IDT and focused IDT (see Figure 5b). The mixing efficiency was observed to be proportional to the square of the applied voltage. Under the same applied voltage, the focused IDT design shows a superior performance compared to the straight IDT design. Good mixing could be achieved within a few tenths of a millisecond. The device provides a mixing efficiency close to 0.9 under an applied peak-to-peak voltage of 80 V, and at a Péclet number as high as 74.4 × 10^3^. Later, Zeng et al. [103] also demonstrated rapid and homogeneous mixing of channel flow at a high flow rate of 5.4 mm/s using a 19.29 MHz focused SAW. Destgeer et al. [104] employed 133.3 MHz-focused SAWs to produce chaotic acoustic streaming flow to mix confluent streams of chemicals in a controlled manner, thereby generating adjustable and rapidly switching gradients in microfluidic channels.

Nam et al. [105] introduced a conductive liquid-based focused SAW (CL-FSAW) device for mixing (see Figure 5c). The PDMS channel contains electrode channels as focused IDTs for focused SAW generation and a main fluidic channel. The electrode channels are designed as focused IDTs with a concentric arc shape with a 60° opening to generate a concentrated acoustic force. The channels contain a serpentine shape with an inlet and outlet for an effective filling process of the conductive liquid. The wavelength of the electrode channels was 800 μm. Therefore, the resonant frequency in the double-electrode IDT mode can be 4.6 MHz or 9.2 MHz. With an applied voltage of 21 V, the mixing efficiency was greater than approximately 97% at a flow rate of 80 μL/min (Pe *=* 1.01 × 10^6^).

Lim et al. [106] introduced a dome-shaped chamber-based SAW (DC-SAW) device for mixing (see Figure 5d). The dome-shaped chamber device with a contact angle of 68° was able to maximize the effect of the SAW transmitted at a refraction angle of about 22°. Enhanced acoustic mixing of two different fluids, namely deionized water and a suspension of fluorescent particles, using 39.6 MHz focused SAWs was achieved in a dome-shaped chamber device. The mixing performance was enhanced with decreasing flow rate and increasing applied voltage. At an applied voltage of 20 V, the total flow rate was 300 μL/min, and the mixing index was above 0.9.

Zhang et al. [107] demonstrated a flexible platform for performing various protocols on the chip. The mixing process was enhanced by acoustic streaming, and the customized delivery of fluid into the mixing chamber was controlled by a single-layer valve (see Figure 5e). Within protocol-specific microfluidic devices, the fluid process flow can be predefined. The added dexterity in terms of fluid delivery potential utilizing this platform allows flexibility to perform various protocols. The optimum operating condition has been identified by evaluating the effect of frequency and power applied on the mixing time. Three frequencies (48.75 MHz, 70.9 MHz, and 130 MHz) were tested, and experiments indicate that the middle frequency (70.9 MHz) has the best performance, resulting from the trade-off between the effects of frequency on attenuation length and body forces. The effect of applied power was tested at the middle frequency, resulting in a reduction of mixing time to 5 s for 153 nL and 1.5 s for 44.3 nL at an excitation power of 28 dBm. On-chip protein crystallization has been conducted to demonstrate the capability of performing biological reactions, resulting in more uniform protein crystal sizes as compared to the case without SAW mixing, demonstrating the need for rapid and efficient mixing.

Besides focused IDTs, SFITs are also used in SAW micromixer designs (see Figure 5f) [108,109]. When an input frequency is applied to an SFIT, only the portion of the IDT that supports the resonant condition is excited, resulting in a smaller aperture for SAWs to propagate compared to a straight IDT with parallel electrodes. Therefore, the lateral position of the excitation wave can be fine-tuned by adjusting the frequency, allowing for more precise control of the mixing region.

### 3.3. Multi-IDT Designs

**Figure 6:**
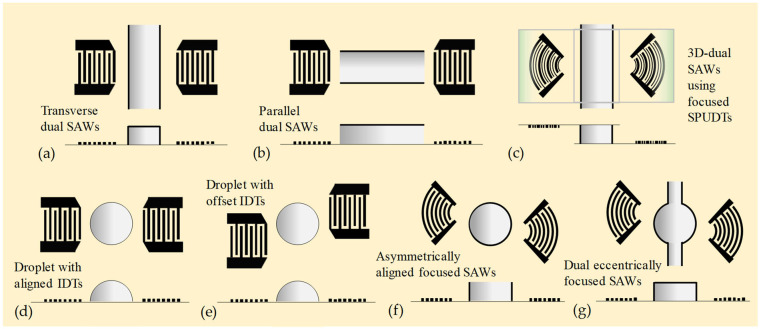
Microfluidic mixing via SAWs generated by multiple IDTs. (**a**) Active mixing technique using dual acoustic streaming field induced by transversely arranged SAWs in a microfluidic channel [110,111]. (**b**) Similar to (a) but using parallel arrangement [110]. (**c**) Mixing technique using three-dimensional dual SAWs (3D-dSAWs) generated from two focused SPUDTs of top and bottom piezoelectric substrates [112]. (**d**) Two aligned IDTs on droplet actuation to achieve efficient mixing of a sessile droplet [113]. (**e**) Similar to (d) but using two offset IDTs [113]. (**f**) Mixing technique using asymmetrically aligned focused SAWs to enhance sensitivity of microarray electrode detection [114]. (**g**) Enhanced micromixing driven by dual eccentrically focused SAWs (DEF-SAWs). The DEF-SAWs are excited by two eccentrically arranged, coplanar, focused IDTs patterned on the surface of a 500 μm-thick LiNbO_3_ substrate [115].

Multiple excitations by multiple IDTs may facilitate the use of the acoustic field more efficiently and accelerate the acoustic mixing [110,111]. Figure 6 shows different layouts of SAW micromixers using multiple IDTs, where Figure 6a,b show two conventional transverse and parallel configurations with dual IDTs. Jo and Guldiken [111] introduced a highly efficient active mixing technique using dual acoustic streaming field induced by SAWs in a microfluidic channel (see Figure 6a). They demonstrated active mixing of a fluorescent dye solution and deionized water in a microfluidic channel using single-acoustic excitation by one IDT, as well as dual excitation by two IDTs. The adopted SAW frequency was 13.3 MHz, and the voltage fed to the IDTs was varied from 28 V to 85 V. Three different flow rates of 10, 50, and 100 μL/min were applied to investigate the effect of the working parameters on the mixing efficiency. Their results indicate that with the same operation parameters, the mixing efficiency with the dual-IDT design increased to 96.7% from 69.8% achievable with the traditional single-IDT design.

Nam and Lim [112] proposed a mixing technique using three-dimensional dual SAWs (3D-dSAWs) generated from two focused SPUDTs of top and bottom piezoelectric substrates (see Figure 6c). By using the 3D-dSAW, internal swirling in a single direction can be induced, which can facilitate more efficient mixing of a channel flow. The working frequency for the used focused SPUDTs was 30 MHz. Their results showed that, with the applied voltage of 14 V, the 3D-dSAW mixing device could achieve 100% mixing efficiency at the flow rate of 50 μL/min, while the mixing efficiency of the traditional single SAW mixer was approximately 38%.

Biroun et al. [113] studied the large deformation of droplet interface actuated by SAWs and explored the complex physics of SAW–droplet interactions and interfacial phenomena. They used a computational interface tracking method based on the coupled level set of the volume of fluid (CLSVOF) approach to simulate the interactions between liquid and acoustic waves and deformation of the liquid–air surface. A dynamic contact angle boundary condition was developed and validated by experimental results to simulate the three-phase contact line dynamics and to study the droplet jetting and internal streaming behaviors by analyzing the energy terms within the liquid medium. The effects of configurations and positions of two IDTs on droplet actuation have been investigated to achieve efficient mixing, separation, and jetting. Results show that two perfectly aligned IDTs are optimal for mixing applications (see Figure 6). In contrast, two offset IDTs are optimal for concentration and separation applications. The maximum jetting velocity and minimum jetting time are achieved by using a pair of aligned IDTs, whereas by using the two offset IDTs (see Figure 6e), effective liquid mixing and jetting are observed, which can be used in bioprinting applications.

Zheng et al. [114] developed a highly sensitive microarray electrochemical-sensing microsystem that integrated a miniature electrochemical microarray sensor and focused SAW micromixers (see Figure 6f). They analyzed the effects of various focusing angles of the focused SAW devices on acoustic wave amplitudes and torques, which determine the acoustic streaming velocity. The electrochemical-sensing microsystem was used to measure the variations of the instantaneous currents of ferrocene carboxylic acid and potassium phosphate buffer solution. The maximum value of the instantaneous current was increased up to 11 times with the applied focused SAWs.

Hsu and Liao [115] proposed enhanced micromixing driven by dual eccentrically focused SAWs (DEF-SAWs), as shown in Figure 6g. The DEF-SAWs are excited by two eccentrically arranged, coplanar, focused interdigital transducers (IDTs) patterned on the surface of a 500 μm-thick LiNbO_3_ substrate. The two focused SAW beams can deliver concentrated acoustic energy into a circular chamber-embedded microchannel to generate enhanced encircling stirring. Compared with using the conventional straight channel, the circular chamber-embedded channel is more suitable for accommodating the encircling circulation flow induced by Eckart streaming and prolongs the working time for mixing. Hence, the joint action of Rayleigh–Schlichting streaming and Eckart streaming achieves high mixing efficiency.

Table 3 summarizes the characteristics of SAW-based micromixers reviewed in this article, including the IDT configuration, microfluidic type, SAW field, and operating frequency, power source, flow rate/fluid volume, and mixing efficiency, to provide a quick overview of the state-of-the-art in SAW-based micromixers.

## 4. Applications

**Figure 7:**
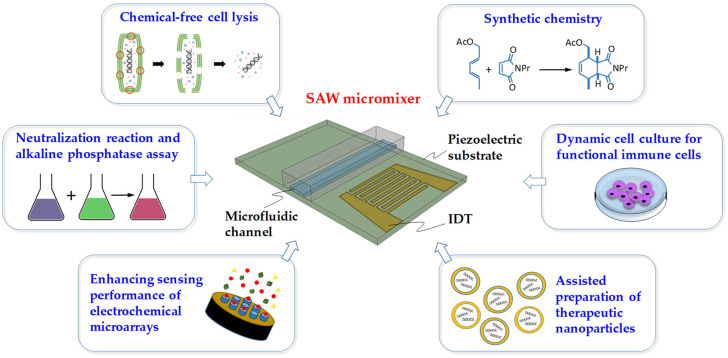
The wide applications of SAW-based micromixers.

Due to their unique features, such as simple structure, good biocompatibility, and high flexibility, SAW micromixers hold great promise in many chemical and biomedical engineering applications, as shown in Figure 7. In the following, we illustrate examples of SAW micromixers in advancing the research fields of chemical synthesis, nanoparticle fabrication, cell culture, biochemical assay, and cell lysis.

Kulkarni et al. [116] demonstrated the use of SAWs as a new modality of synthetic chemistry to input energy into droplet-scale chemical reactions. Reaction mixtures can be simply applied as drops onto an appropriate surface and reacted for short periods of time. For the application procedure, a small drop of the reaction mixture was placed on the LiNbO_3_ substrate between the IDTs and completely within the path of the SAW radiation. When SAW is actuated, the drop will vibrate vigorously if the viscosity of the reaction mixture is low. If the viscosity is significantly higher than that of typical organic solvents, an internal streaming pattern can be observed. SAW irradiation continued until the reaction was complete. The reactions can be conducted neat or in a relatively nonvolatile solvent. In general, the reactions are fast, yields are high, and the products are clean. When combined with the well-established ability of SAWs to actuate drops, the results lay the foundation for a powerful approach to the manipulation and processing of chemicals.

Westerhausen et al. [109] demonstrated the applicability and advantages of SAW-assisted fabrication of therapeutic nanoparticles (TNPs) and elucidated the role of controllable parameters in the mixing process of aqueous solutions, as well as other solvents such as ethanol and isopropanol. They showed that the ratio of the mainstream velocity along the channel axis to the SAW-induced flow velocity perpendicular to that axis is most important and can be tuned by increasing the SAW power or decreasing the flow velocity. Mixing without SAW produced larger particles compared to hand mixing, whereas particle formation with SAW assistance produced particles of the desired size comparable to hand mixing. Furthermore, this technique enhances the formation of more complex mono-nucleic acid/lipid particles (mNALPs), resulting in increased encapsulation and improved reproducibility. SAW-assisted fabrication of therapeutic nanoparticles offers unique, promising, and controllable technology for nanomedicine, especially for particle systems that are very sensitive to preparation conditions.

Kim et al. [117] investigated the cytotoxic response of natural killer (NK) cells in a microreactor to SAWs. Rayleigh-type SAWs allow the dynamic stimulation of functional immune cells in a noncontact and rotor-free manner. The SAW microreactor enables a dynamic cell culture to be set up in a miniaturized system while maintaining the performance of agitating media. The SAW system creates acoustic streaming flow in the cylindrical microreactor and applies flow-induced shear stress to the cells. The suspended NK cells are found not to be damaged by the SAW operation. Suspended NK cell aggregates subjected to an SAW treatment show increased intracellular Ca^2+^ concentrations. Simultaneously treating the NK cells with SAWs and protein kinase C activator enhances the lysosomal protein expression of the cells and the cell-mediated cytotoxicity against target tumor cells. These have shown that an acoustically actuated system allows dynamic cell culture without cell damage and further alters cytotoxicity-related cellular activities.

Bai et al. [118] developed an SAW-assisted micromixer with active temperature control to balance the thermal effect of the SAW for optimized temperature management of the reactant fluid inside the microchannel during the SAW-assisted microreaction. The use of this system ensures efficient and full SAW-assisted microreaction. Two typical biochemical reactions, the neutralization reaction and the alkaline phosphatase (ALP) assay, have been utilized to verify the effectiveness of the temperature control of the SAW micromixer. The neutralization reaction was performed on this device with different input voltages. There was a significant process intensification under the impact of SAW. Additionally, the temperature-sensitive ALP catalytic reaction was conducted on different states of this device, i.e., with and without thermal control. The substrate has been fully catalyzed under the maximum acoustic streaming at 37 °C, along with temperature control, while the microreaction barely happened at all without thermal control. The study demonstrates the potential of this new SAW-assisted micromixer for various temperature-sensitive biomedical applications.

Zheng et al. [114] demonstrated a highly sensitive microarray electrochemical detection platform integrated with focused SAW mixing technology. The wavelength of high-frequency SAW is comparable to the size of the flow channel of microfluidic systems, which can efficiently mix fluids in microfluidic systems. The focused SAW mixing method shows an excellent ability to enhance microfluidic mixing and improve the sensing accuracy of electrochemical sensors. The obtained value of the focused SAW-enhanced array microelectrode electrochemical sensor was 4.3 μA, which is 11 times higher than that of the array microelectrode electrochemical sensor without applying focused SAWs. The focused SAW-enhanced electrochemical detection platform achieves an excellent detection accuracy and well-integrated performance, providing great potential for the development of microscale sensing platforms to detect food, environmental, and biological samples.

Cha et al. [100] demonstrated chemical-free acousto-microfluidic cell lysis at high throughput. The lysed red blood cells (RBCs) were visibly and quantitatively verified, and in-depth characterization of lysis efficiency was conducted under a controllable intensity of the acoustic streaming flow (ASF) mixing. Consequently, not only high lysis efficiency (>90%) but also controllable lysis could be realized in a chemical-free and continuous manner. Based on their findings, efficient ultrasonic SAW micromixers can be a promising approach for microfluidic mixing and can be employed in various chemical and biomedical applications.

Table 4 summarizes these recent advances in SAW-based micromixers for microfluidic applications reviewed in this section.

## 5. Conclusions and Prospects

This review provided a systematic overview of the latest research and development of SAW-based micromixers. Specifically, we have explained the mixing behavior in a microfluidic environment, discussed the working principles and theoretical approaches of SAW devices, IDT designs, and acoustofluidics, classified SAW-based micromixers into three different configurations (including single straight IDT, single non-straight IDT, and multiple IDTs), and highlighted several mixing demonstrations in chemical synthesis, nanoparticle fabrication, cell culture, biochemical analysis, and cell lysis. Due to its advantages over other micromixing techniques, such as label-free operation, flexible control, large and contactless forces, fast-reaction kinetics, robust mixing, and good biocompatibility, SAW micromixers have been demonstrated to have great potential to advance both fundamental and applied research. Although prototyping work has been done and great progress has been made, many challenges remain in the accessibility and affordability of SAW-based micromixers. In conclusion, future research on SAW-based micromixing should focus on improving operability and efficiency [119]. First, feasible approaches are needed to improve the electromechanical conversion efficiency from electrical energy input to SAW energy and reduce the heating effects to address these generally inherent limitations of SAW micromixers. Second, structural and SAW field optimization can improve acoustofluidic interactions, throughput, and mixing rates, helping SAW-based micro-mixing chips move from laboratory demonstrations to practical applications to better meet clinical and industrial needs. Third, focusing on lab-on-a-chip integration for practical microfluidic analytical applications can make SAW micromixing technology applicable to a wider range of microfluidics fields and reduce the requirements for chip manufacturing and operation. Achieving these goals will enable SAW-based mixing technology to have a wider range of application scenarios.

## Figures and Tables

**Table 1:** A schematic comparison of passive and active micromixers [25,27,29].

Type	Example Techniques	Mixing Efficiency	Mixing Time	Mixing Length	Potential Pros and Cons
Passive	Split and recombination	80–99%	-	10^3^–10^5^ μm	No power supply required; Longer channels; Complex channel geometries; Expensive 3D channels
	Surface patterning				
	Hydrodynamic focusing				
	Serpentine channel				
	Obstacles in flow				
Active	Acoustic	80–99%	10^–3^–10^1^ s	-	External power supply required; Simpler channel geometries; Rapid and controllable mixing; Labeled fluids
	Electrical				
	Magnetic				
	Pressure				
	Thermal				

**Table 2:** A comparison of active micromixing techniques [43,44].

Technique	Advantages	Limitation	Key to Improvement
Acoustic	Rapid mixing; easy operation; direct mechanical force	Low throughput; high cost; high power consumption	Improve mixing performance by enhancing fluid-structure interaction
Electrical	Efficiently used at low voltages; effective for short mixing lengths	Requires integrated electrodes and conductive liquids	Reduce the influence and dependence on conductive liquids
Magnetic	Efficient mixing with precise control; versatile	Requires magnetic materials or magnetic labels in samples/reagents	Enhance magnetic field strength for microscale applications
Pressure	Easy to implement using micropumps or electric fields	Requires fine-tuning for optimal mixing	Optimize operating pulsation parameters driven by the integrated pumps
Thermal	Easy to integrate into the microfluidic devices	Requires heaters; heating effects on samples/reagents	Reduce heating influences on samples/reagents

**Table 3:** Summary of the SAW-based micromixers reviewed in this article.

Design	Microfluidic Type	Acoustic Field and Frequency	Power Source	Flow Rate/Fluid Vol.	Mixing Efficiency	Ref.
Straight IDT	Channel flow	SAW and APW, 10 MHz	30 dBm	20 μL/min	-	[94]
	Fluid well	SAW, 20 MHz	<1.6 W	2.5 μL	-	[95]
	Sessile droplet	SAW, 47.8–1107 MHz	-	0.6–58 nL	-	[96]
	Confined droplet	SAW, 27.5 MHz	28.5 dBm	2 μL	-	[97]
	Channel flow	SAW to Lamb wave, 50 and 100 MHz	50 V	1 μL/min	80%	[98]
	Channel flow	SAW, 140 MHz	12 V	50 μL/min	~100%	[99]
	Sessile droplet	SAW, 29 MHz	0.5 W	2 μL	~100%	[100]
Non-straight IDT	Sessile droplet	Focused SAW, 30 MHz	<1 W	2 μL	-	[101]
	Channel flow	Focused SAW, 13 MHz	80 V	10 mL/h	88%	[102]
	Channel flow	Focused SAW, 19.29 MHz	25 V	2700 μm/s	-	[103]
	Channel flow	Focused SAW, 133.3 MHz	0–17.9 V	1100 μL/h	-	[104]
	Channel flow	Focused SAW, 9.2 MHz	21 V	<120 μL/min	>90%	[105]
	Channel flow	Focused SAW, 39.6 MHz	20 V	<300 μL/min	>90%	[106]
	Chamber	Focused SAW, 48.75, 70.9, and 130 MHz	28 dBm	44.3, 153 nL	-	[107]
	Channel flow	SAW excited by a SFIT, 79.5–82.5 MHz	25 dBm	0.2 mL/h	~100%	[109]
Multiple IDTs	Channel flow	Dual SAW, 9.6 MHz	35 V	513 μm/s	94%	[110]
	Channel flow	Dual SAW, 13.3 MHz	85 V	10 μL/min	96.7%	[111]
	Channel flow	3D dual focused SAW, 30 MHz	<18 V	<120 μL/min	>90%	[112]
	Sessile droplet	Offset dual SAW, 66.2 MHz	12 W	2 μL	-	[113]
	Fluid well	Asymmetrically aligned focused SAW, 19.3 MHz	15 V	40 μL	-	[114]
	Channel flow	Dual eccentrically focused SAW, 9.65 MHz	12.5 V	7 μL/min	96%	[115]

**Table 4:** Summary of recent advances in SAW-based micromixers for microfluidics applications.

IDT Design	Structure Characteristics	Application	Ref.
Two straight IDTs	A 40 mL drop of reaction mixture on a piezoelectric substrate with IDTs at either end of the device connected to a power source.	Synthetic chemistry	[116]
An SFIT	Two equal-sized inlets of the Y-shaped elastomeric microchannel converge into a rectangular main channel with a width of 200 μm. The IDT is patterned on one side of the channel.	Assisted preparation of therapeutic nanoparticles	[109]
A straight IDT	A microreactor system including an open-top fluid well and an IDT on a LiNbO_3_ substrate.	Dynamic cell culture for functional immune cells	[117]
A focused IDT	Microchannel with integrated temperature control unit, which consists of a temperature sensor under the microchannel and a Peltier cooler under the microchip.	Neutralization reaction and alkaline phosphatase assay	[118]
Two focused IDTs	Two focused IDTs are asymmetrically distributed on both sides of a ring structure (a fluid well), producing two focused SAWs propagating in opposite directions, which generate acoustic streaming.	Enhancing the sensing performance of electrochemical microarrays	[114]
A straight IDT	The device comprises a piezoelectric LiNbO_3_ substrate with Cr/Au patterned IDT, and a SiO_2_ layer is deposited on the substrate to protect the electrodes. A straight microchannel with two serial inlets and one outlet was bonded to the substrate.	Chemical-free cell lysis	[100]

## Data Availability

The data that support the findings of this study are available within the article.
